# Secular trends in the prevalence of low risk factor burden for cardiovascular disease according to obesity status among Chinese adults, 1993–2009

**DOI:** 10.1186/1471-2458-14-961

**Published:** 2014-09-16

**Authors:** Tingting Du, Xingxing Sun, Ping Yin, Gang Yuan, Muxun Zhang, Xinrong Zhou, Xuefeng Yu

**Affiliations:** Department of Endocrinology, Tongji Hospital, Tongji Medical College, Huazhong University of Science and Technology, 1095#, Jiefang Road, Wuhan, 430030 China; Department of Anesthesiology, School of Stomatology, Fourth Military Medical University, Xi’an, 710032 China; Department of Epidemiology and Biostatistics, School of Public Health, Tongji Medical College, Huazhong University of Science and Technology, Wuhan, 430030 China

**Keywords:** Cardiovascular risk factors, Trends, Waist circumference, Body mass index, Epidemiology

## Abstract

**Background:**

Cardiovascular disease (CVD) and obesity are now common among Chinese. We aimed to examine secular trends in the prevalence of low risk profile and to examine whether comparable changes in the prevalence of low risk profile across waist circumference (WC) groups and body mass index (BMI) categories have occurred.

**Methods:**

We used data from the nationwide China Health and Nutrition Survey conducted in 1993, 1997, 2000, 2004, 2006, and 2009. There were 7274, 8368, 9369, 8948, 8786, and 9278 participants included in the analyses across the six study periods. We created an index of low risk factor burden from the following variables: not currently smoking, BMI < 25 kg/m^2^, WC < 90/80 cm in men/women, untreated systolic/diastolic blood pressure < 120/80 mmHg, and not having been previously diagnosed with diabetes.

**Results:**

During the period of 1993–2009, the age-adjusted prevalence of low risk profile decreased from 16.2 to 11.5% among men and from 46.3 to 34.6% among women (both P < 0.001); Similar significant trends were observed in all age groups, rural/urban settings, education groups, WC status and BMI categories. The change in the prevalence of low risk profile was more striking among obese persons (P for interaction terms cohort *BMI were < 0.001). In 2009, 2.0 and 25.6% among central obese men and women had a low risk profile; Of note, was that 0.1 and 0.3% general obese men and women had a low risk profile.

**Conclusions:**

The prevalence of low risk profile declined considerably over the past 17 years in all demographic groups, WC status, and BMI categories. Public health prevention strategies are urgently needed.

**Electronic supplementary material:**

The online version of this article (doi:10.1186/1471-2458-14-961) contains supplementary material, which is available to authorized users.

## Background

Over the past 2 decades, the mortality from cardiovascular disease (CVD) have declined in the United States [1] and most Western European countries [2]. By contrast, CVD mortality rates are still rising in China [3]. With the economic development and accelerated pace of nutrition transition in China [4], the number of people suffering from prehypertension/hypertension, prediabetes/diabetes, and/or dyslipidemia have been growing larger [5–8]. This phenomenon indicates a further increase in CVD mortality rates in the near future. Estimating the secular trends in the prevalence of major CVD risk factors can partly explain the trends in CVD mortality rates [1, 9]. Besides, monitoring the trends in the prevalence of CVD risk factors in the population may provide information on knowing how much progress in reducing the CVD mortality remains to be potentially achieved, and offer an opportunity for intervention in clinical practice and public health campaigns. However, little information is available on recent national estimates of trends in the prevalence of CVD risk factors in China. In addition, the prevalence of central obesity assessed by elevated waist circumference (WC) and general obesity assessed by elevated body mass index (BMI), important risk factors for CVD, have increased dramatically in China over the past 2 decades [10]. The association between obesity trends and other CVD risk factors is scant in China. Accumulating evidence showed that individuals with low levels of cardiovascular risk factors experience low rates of subsequent cardiovascular mortality [11–13]. Hence, we took advantage of the data from currently available waves of the China Health and Nutrition Survey (CHNS) to describe the secular trends in the prevalence of low risk factor burden and further to assess whether comparable changes in the prevalence of low risk factor burden across WC groups and BMI categories have occurred.

## Methods

### Study design

The CHNS is the only large-scale longitudinal, household-based survey in China [14]. The CHNS was designed to represent a set of large provinces with significant variation in terms of geography, economic development, and health status, covering approximately 56% of China’s population, including Liaoning, Heilongjiang, Jiangsu, Shandong, Henan, Hubei, Hunan, Guangxi, and Guizhou. The CHNS waves were conducted in 1989, 1991, 1993, 1997, 2000, 2004, 2006, 2009 and 2011. For each wave, a stratified multistage, random cluster process was employed to draw study sample from each of the nine provinces. Counties in the nine provinces were stratified by income (low, middle and high) and a weighted sampling scheme was used to select randomly four counties in each province. Full details of the CHNS have been described elsewhere [14]. Each participant provided a written informed consent and the study was approved by the institutional review committees of the University of North Carolina at Chapel Hill, the National Institute of Nutrition and Food Safety, Chinese Center for Disease Control and Prevention, and the China-Japan Friendship Hospital, Ministry of Health.

### Study population

Since WC was collected initially in 1993, we examined data from CHNS: 1993, 1997, 2000, 2004, 2006, and 2009. All participants were asked to complete a structured questionnaire which provided information on age, sex, rural/urban settings, educational attainment, smoking habits, histories of current and previous illness, and medical treatment. There were 8,321, 10,551, 9,688, 9,813, 9,752 and 10,038 participants included in 1993, 1997, 2000, 2004, 2006 and 2009 surveys, respectively. Participants were eligible in the present analysis if they were 18 years or older. Exclusion criteria included pregnancy, no information on age, WC, weight, height or blood pressure. The analytic sample sizes were 7274 (87.4%) for 1993, 8368 (79.3%) for 1997, 9369 (96.7%) for 2000, 8948 (91.2%) for 2004, 8786 (90.1%) for 2006, and 9278 (92.4%) for 2009. The percentages of women and rural residents remained relatively stable during the period covered by the surveys (P for trend > 0.05).

### Measurements

Weight, height, WC and blood pressure (BP) were measured following standardized protocols from the World Health Organization (WHO) [15]. Weight was measured with the participants wearing light clothing and height was measured without shoes. BMI was calculated as weight (in kilograms) divided by the square of height (in meters). WC was measured with an inelastic tape at a midpoint between the bottom of the rib cage and the top of the iliac crest at the end of exhalation. Seated systolic/diastolic BP was measured by trained technicians in triplicate after a 10-min rest, using mercury manometers. The three readings were averaged as the BP values in our data analysis. All physical examinations were performed at the same location and followed the same protocol at each study visit.

### Definitions

#### Low risk factor burden

According to the American Heart Association’s (AHA) definition of “ideal cardiovascular health metrics” [16] and results from previous studies [17–19], we created an index of low risk factor burden from the following variables: not currently smoking, BMI < 25 kg/m^2^, WC < 90 cm for men and < 80 cm for women, untreated systolic BP < 120 mmHg and diastolic BP < 80 mmHg, and not having been previously diagnosed with diabetes. The simultaneous presence of the 5 health metrics mentioned above represents low risk profile. When we examined trends in low risk factor burden by WC groups, we defined the low risk profile as the simultaneous presence of not currently smoking, BMI < 25 kg/m^2^, untreated systolic/diastolic BP < 120/80 mmHg, and not having been previously diagnosed with diabetes. Similarly, when we examined trends in low risk factor burden by BMI categories, we defined the low risk profile as the simultaneous presence of not currently smoking, WC < 90/80 cm for men/women, untreated systolic/diastolic BP < 120/80 mmHg, and not having been previously diagnosed with diabetes. Not currently smoking was defined as having not ever smoked or having smoked less than 100 cigarettes, but not at the time of the interview.

#### General obesity and central obesity

According to WHO suggestions [20], normal weight is defined as BMI < 25 kg/m^2^, overweight is defined as BMI of 25–29.9 kg/m^2^, and general obesity is defined as BMI ≥ 30 kg/m^2^. According to WHO suggestions for Asians [19], central obesity is defined as WC ≥ 90 cm for men and ≥ 80 cm for women.

### Statistical analysis

All statistical analyses were conducted using SPSS software (version 12.0 for windows; SPSS, Chicago, IL, USA). Categorical variables were presented as percentages. Analyses were stratified by sex, age groups (18–44 years, 45–64 years, and 65–118 years), urban/rural settings, and educational attainment (less than high school, high school, and university). Because the mean age increased successively from 1993 to 2009 (P < 0.001), to maximize the comparability between surveys, all survey data (i.e. the prevalence of low risk profile) were age-standardized to the age distribution of 2000 census of the Chinese adult population by the direct method. Trends in the prevalence of low risk profile and its components from 1993 to 2009 were assessed by Cochran-Armitage trend testing. To assess if changes between the first and last surveys differed by WC group or BMI categories, logistic regression analysis was utilized to examine potential interaction effects between cohort and WC status or between cohort and BMI categories. A two-tailed *P* value of < 0.05 was considered to be significant.

## Results

**Figure 1:**
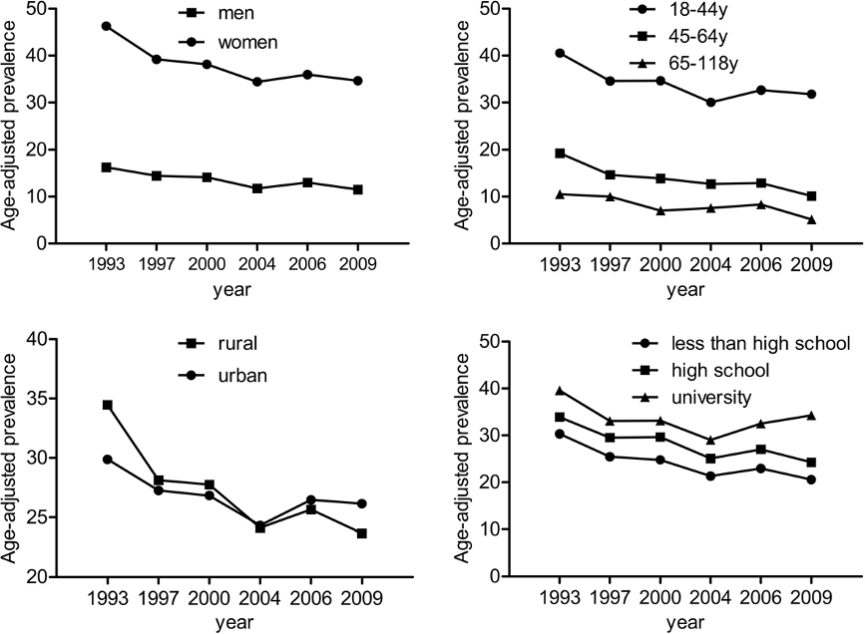
**Trends in the age-adjusted prevalence of low risk profile by sex, age, rural/urban setting, and educational attainment: the CHNS 1993–2009.** The low risk profile was defined as the simultaneous presence of not currently smoking, body mass index < 25 kg/m^2^, waist circumference < 90/80 cm in men/women, untreated systolic/diastolic blood pressure < 120/80 mmHg, and not having been previously diagnosed with diabetes.

Detailed information on trends in the age-standardized prevalence of low risk profile across the 6 consecutive CHNS waves were illustrated by sex, age, rural/urban settings, and educational attainment in Figure 1. Although the prevalence of low risk profile among women approximately tripled those among men in each survey (*P* < 0.001), similar significant trends were observed in men and women, with the prevalence of low risk profile decreased from 16.2 in 1993 to 11.5% in 2009 among men, and from 46.3 to 34.6% among women (both *P* for nonlinear trend < 0.001). The prevalence of low risk profile decreased over the study period in all age groups, rural/urban settings, and education groups (all *P* for trend < 0.001). Notably, the change in the prevalence of low risk profile was more striking among rural residents than that among urban counterparts (*P* < 0.001 for interaction terms cohort * rural/urban settings). For each survey, the prevalence of low risk profile declined linearly with age (*P* for linear trend < 0.001 for each survey); In addition, the prevalence of low risk profile was much higher among participants in the highest education group (university) than those among participants in middle education group (high school) or those in the lowest education group (less than high school) (*P* < 0 .001 for each survey).

Trends in the age-adjusted prevalence of 5 components of the low risk profile were assessed separately in Additional file 1. The 5 components did not move uniformly during the period 1993–2009, with the trend in the prevalence of not currently smoking increased (*P* < 0.001), and the trends in the prevalence of other low risk factors decreased over time (all *P* < 0.05). Similar directions of the trends for each individual component of the low risk profile were observed in all demographic groups (all *P* < 0.05). The age-adjusted prevalence of not currently smoking and untreated SBP/DBP < 120/80 mmHg among women significantly exceeded those among men in each survey (*P* < 0.001 for each survey).

Between 1993 and 2009, although the rate of not currently smoking among men with WC < 90 cm increased (Table 1), the interaction between WC and cohort on the proportion of not currently smoking was not statistically significant (*P* = 0.18). Although the prevalence of BMI < 25 kg/m^2^, untreated systolic/diastolic BP < 120/80 mmHg, not having been previously diagnosed with diabetes, and the low risk profile (the simultaneous presence of not currently smoking, BMI < 25 kg/m^2^, untreated systolic/diastolic BP < 120/80 mmHg, and not having been previously diagnosed with diabetes) were higher among cohorts of men with WC < 90 cm, we found significantly decrease in rates of them irrespective of WC category (*P* for interaction terms cohort * WC = 0.39, 0.32, 0.92, and 0.64, respectively) (Table 1); The age-adjusted prevalence of the low risk profile decreased from 17.5 in 1993 to 15.0% in 2009 among normal-WC men and from 4.2 in 1993 to 2.0% in 2009 among central obese men (both *P* < 0.001). There was a marked decrease in the rate of BMI < 25 kg/m^2^ among women with WC ≥ 80 cm, however, cohort * WC interaction terms comparing the first and sixth surveys were not statistically significant (P for interaction terms cohort * WC = 0.45), indicating that secular declines in the rate of BMI < 25 kg/m^2^ did not differ by WC group. Although the prevalence of not currently smoking, untreated systolic/diastolic BP < 120/80 mmHg, not having been previously diagnosed with diabetes, and the low risk profile were higher among women with WC < 80 cm, significantly decrease in rates of them, irrespective of WC category were noted (P for interaction terms cohort * WC = 0.94, 0.45, 0.95, and 0.06, respectively). The age-adjusted prevalence of low risk profile decreased from 60.9% in 1993 to 55.0% in 2009 among normal-WC women and from 30.7% in 1993 to 25.6% in 2009 among central obese women (both *P* < 0.001).

**Table 1:** Trends in prevalence of low risk profile and its components by waist circumference (WC) status*

		1993	1997	2000	2004	2006	2009	Δ ^†^	P for trend ^‡^	P ^§^
Men	The simultaneous presence of 4 health metrics^‖^ (%)									
	WC < 90 cm	17.5	16.4	16.9	14.5	16.5	15.0	-2.5	<0.001	0.64
	WC ≥90 cm	4.2	4.7	3.7	5.1	3.4	2.0	-2.2	0.004	
	Not currently smoking (%)									
	WC < 90 cm	37.7	40.9	42.6	43.4	45.7	43.8	6.1	<0.001	0.18
	WC ≥90 cm	42.9	46.5	50.1	52.0	50.7	50.6	7.7	0.140	
	Body mass index <25 kg/m^2^ (%)									
	WC < 90 cm	92.8	92.5	90.1	87.8	87.8	89.0	-3.8	0.0151	0.39
	WC ≥90 cm	44.6	28.7	32.7	35.7	28.4	26.8	-17.8	<0.001	
	Untreated systolic/diastolic blood pressure <120/80 mmHg (%)									
	WC < 90 cm	50.0	42.1	41.2	37.3	38.6	37.2	-12.8	<0.001	0.32
	WC ≥90 cm	23.2	19.1	18.0	16.5	19.1	17.8	-5.4	0.006	
	Not having been previously diagnosed with diabetes (%)									
	WC < 90 cm	100.0	99.7	99.6	99.3	98.8	98.7	-1.3	<0.001	0.92
	WC ≥90 cm	99.9	99.0	99.1	97.9	97.3	96.3	-3.6	<0.001	
Women	The simultaneous presence of 4 health metrics^‖^ (%)									
	WC < 80 cm	60.9	53.3	54.8	52.3	55.1	55.0	-5.9	<0.001	0.06
	WC ≥80 cm	30.7	24.0	25.0	24.8	25.2	25.6	-5.1	<0.001	
	Not currently smoking (%)									
	WC < 80 cm	95.7	95.7	96.4	96.9	97.2	97.6	1.9	0.0209	0.94
	WC ≥80 cm	95.8	95.6	95.5	96.8	97.6	97.6	1.8	<0.001	
	Body mass index <25 kg/m^2^ (%)									
	WC <80 cm	95.0	94.9	93.8	94.4	94.0	95.7	0.7	0.158	0.45
	WC ≥80 cm	59.4	54.6	52.7	51.4	53.9	55.0	-4.4	0.0126	
	Untreated systolic/diastolic blood pressure <120/80 mmHg (%)									
	WC < 80 cm	65.1	57.5	59.2	56.2	58.9	57.8	-7.3	<0.001	0.21
	WC ≥80 cm	47.6	40.1	42.0	43.0	40.1	39.2	-8.4	<0.001	
	Not having been previously diagnosed with diabetes (%)									
	WC < 80 cm	100.0	99.5	99.5	99.3	99.4	99.1	-0.9	<0.001	0.95
	WC ≥80 cm	100.0	99.2	98.8	98.7	98.1	98.0	-2.0	<0.001	

*Estimates are weighted to be representative of the Chinese population aged 18 to 118 years.

^†^The sixth survey-the first survey (2009–1993);

^‡^Trends in prevalence of low risk profile and its components from 1993 to 2009 were assessed by Cochran–Armitage trend testing;

^§^P for survey * WC interaction terms comparing the first and sixth surveys;

^‖^The simultaneous presence of not currently smoking, BMI <25 kg/m^2^, untreated systolic/diastolic BP < 120/80 mmHg, and not having been previously diagnosed with diabetes.

To explore the contribution of body weight to declines in the prevalence of low risk profile and its components, we examined the prevalence of low risk profile and its components by BMI category (Table 2). Between 1993 and 2009, the age-adjusted prevalence of WC < 90/80 cm for men/women, untreated systolic/diastolic BP < 120/80 mmHg, not having been previously diagnosed with diabetes, and the low risk profile (the simultaneous presence of not currently smoking, WC < 90/80 cm for men/women, untreated systolic/diastolic BP < 120/80 mmHg, and not having been previously diagnosed with diabetes) decreased among all BMI groups for both men and women. However, most of the absolute reductions in the prevalence of low risk profile and its components occurred among the general obese group (all *P* for interaction terms cohort * BMI < 0.05). The prevalence of the low risk profile decreased from 11.8 in 1993 to 0.1% in 2009 among obese men and from 21.4 in 1993 to 0.3% in 2009 among obese women (both *P* < 0.001).

**Table 2:** Trends in prevalence of low risk profile and its components by body mass index (BMI) categories*

		1993	1997	2000	2004	2006	2009	Δ ^†^	P for trend ^‡^	P ^§^
Men	The simultaneous presence of 4 health metrics^‖^ (%)									
	BMI < 25 kg/m^2^	18.2	16.9	17.3	15.0	17.1	15.2	-3.0	<0.0001	0.0044
	BMI 25–29.9 kg/m^2^	6.8	4.1	8.2	5.0	5.8	4.4	-2.4	0.0427	
	BMI ≥ 30 kg/m^2^	11.8	11.1	2.3	3.7	3.2	0.1	-11.7	<0.0001	
	Not currently smoking (%)									
	BMI < 25 kg/m^2^	37.8	40.8	42.6	42.9	44.4	42.7	4.9	0.0002	0.1098
	BMI 25–29.9 kg/m^2^	41.6	47.3	50.5	51.0	53.4	53.3	11.7	<0.0001	
	BMI ≥ 30 kg/m^2^	50.8	55.1	47.3	53.4	55.9	49.7	-1.1	0.1338	
	Waist circumference < 90 cm (%)									
	BMI < 25 kg/m^2^	95.6	95.4	92.2	89.9	91.1	89.6	-6.0	<0.0001	0.0009
	BMI 25–29.9 kg/m^2^	58.1	39.9	43.9	42.5	38.5	32.4	-25.7	<0.0001	
	BMI ≥ 30 kg/m^2^	64.0	48.4	17.3	26.0	19.3	3.7	-60.3	<0.0001	
	Untreated systolic/diastolic blood pressure <120/80 mmHg (%)									
	BMI < 25 kg/m^2^	50.0	42.7	41.0	37.6	39.5	38.0	-12.0	<0.0001	0.0391
	BMI 25–29.9 kg/m^2^	29.0	19.5	25.7	17.1	21.6	17.9	-11.1	<0.0001	
	BMI ≥ 30 kg/m^2^	42.6	22.9	14.1	13.1	12.5	9.2	-33.4	<0.0001	
	Not having been previously diagnosed with diabetes (%)									
	BMI < 25 kg/m^2^	100.0	97.0	93.5	99.0	98.6	98.7	-1.3	<0.0001	< 0.001
	BMI 25–29.9 kg/m^2^	99.2	96.6	93.7	97.9	98.0	96.4	-2.8	<0.0001	
	BMI ≥ 30 kg/m^2^	99.9	98.5	93.0	92.8	98.0	91.6	-8.3	0.0002	
Women	The simultaneous presence of 4 health metrics^‖^ (%)									
	BMI < 25 kg/m^2^	53.5	46.6	47.0	42.9	44.6	42.6	-10.9	<0.0001	0.0013
	BMI 25–29.9 kg/m^2^	15.6	15.1	10.6	10.0	9.7	9.1	-5.9	<0.0001	
	BMI ≥ 30 kg/m^2^	21.4	10.7	11.0	16.3	5.8	0.3	-21.1	<0.0001	
	Not currently smoking (%)									
	BMI < 25 kg/m^2^	95.8	95.7	96.1	96.9	97.7	97.7	1.9	0.0021	0.9113
	BMI 25–29.9 kg/m^2^	96.4	95.5	95.2	96.9	97.7	97.8	1.4	0.0168	
	BMI ≥ 30 kg/m^2^	89.8	92.7	96.3	96.2	88.7	82.2	-7.6	0.4429	
	Waist circumference < 80 cm (%)									
	BMI < 25 kg/m^2^	80.7	80.2	76.2	74.0	72.6	69.1	-11.6	<0.0001	0.0035
	BMI 25–29.9 kg/m^2^	27.5	25.5	23.8	15.4	16.3	14.1	-13.4	<0.0001	
	BMI ≥ 30 kg/m^2^	35.2	16.0	27.5	20.7	20.0	0.7	-34.5	<0.0001	
	Untreated systolic/diastolic blood pressure <120/80 mmHg (%)									
	BMI < 25 kg/m^2^	63.3	55.7	58.0	54.8	56.4	54.6	-8.7	<0.0001	0.0029
	BMI 25–29.9 kg/m^2^	45.9	40.1	36.7	41.9	37.6	37.9	-8.0	<0.0001	
	BMI ≥ 30 kg/m^2^	36.9	30.2	31.3	49.7	18.3	15.1	-21.8	0.0002	
	Not having been previously diagnosed with diabetes (%)									
	BMI < 25 kg/m^2^	100.0	96.7	95.7	98.9	98.9	98.7	-1.3	<0.0001	< 0.001
	BMI 25–29.9 kg/m^2^	99.4	96.2	94.3	98.2	98.2	98.0	-1.4	<0.0001	
	BMI ≥ 30 kg/m^2^	95.8	89.1	93.8	97.2	92.3	79.7	-16.1	<0.0001	

*Estimates are weighted to be representative of the Chinese population aged 18 to 118 years.

^†^The sixth survey-the first survey (2009–1993);

^‡^Trends in prevalence of low risk factor profile and its components from 1993 to 2009 were assessed by Cochran–Armitage trend testing;

^§^P for survey * BMI interaction terms comparing the first and sixth surveys;

^‖^The simultaneous presence of not currently smoking, WC < 90/80 cm, untreated systolic/diastolic BP < 120/80 mmHg, and not having been previously diagnosed with diabetes.

## Discussion

In the present study, the prevalence of low risk profile and its components decreased significantly during the period covered by the surveys irrespective of demographic groups, WC status, and BMI categories. In 2009, only 11.5% of men and 34.6% of women had a low risk profile. In addition, the proportion of low risk profile and its components was significantly lower in central obese persons, with 2.0% and 25.6% of central obese men and women had a low risk profile, respectively; Furthermore, absolute reductions in the prevalence of low risk profile and its components between 1993 and 2009 were greater for obese persons, with 0.1% and 0.3% of general obese men and women had a low risk profile, respectively. Our results emphasize the great potential for preventing an unexpectedly huge burden of CVD that remains to be realized in China. This is, to our best knowledge, the first Chinese population-based long-term study to show secular trends in the prevalence of low risk profile and its components.

Few other studies on estimates of point prevalence of CVD risk factors in nationally representative Chinese population were available [21–23]. All these studies examined 5 modifiable CVD risk factors, namely smoking, overweight/obesity, systolic/diastolic BP ≥ 140/90 mmHg, dyslipidemia, and diabetes, and yielded estimates of the rates of low risk factor burden ranging from 7.5% to 10.5% among men and 19.4% to 28.7% among women. Another 2 community-based studies examined 7 modifiable CVD risk factors, namely inadequate physical activity, unhealthy diet, overweight/obesity, systolic/diastolic BP ≥ 140/90 mmHg, dyslipidemia, and hyperglycemia. These two studies showed lower rates of low risk factor burden, with the prevalence ranging from 0.06 to 2.7% among men and 0.26 to 6.9% among women [24, 25]. By contrast, our present study saw a relatively higher prevalence of low risk factor burden, with the prevalence of 11.5% among men and 34.6% among women in 2009. One possible explanation for this issue may be the fact that self-reported diabetes is not a reliable measure as it suggests an underestimation of the diabetes epidemic. Another explanation might be that we covered no information on physical activity, diet, and the lipid profile. However, we extended the CVD risk factors to cover central obesity in view of the independent predictive value of excessive WC for CVD [18, 26]. Including normal WC as a potential marker for the definition of low risk profile may be nondiscriminatory in the identification of individuals with low risk profile as BMI correlated well with WC. However, results of our recent study showed that among those identified to have general or central obesity based on a combination of BMI ≥ 28 kg/m^2^ and WC ≥ 90/80 cm, approximately two thirds of individuals with obesity would be missed if WC was not measured [27]. Hence, we included normal WC as a marker for the definition of low risk profile. Furthermore, more stringent systolic/diastolic BP < 120/80 mmHg cut points rather than the systolic/diastolic BP < 140/90 mmHg cut points were applied in our current study to define the low risk profile in accordance with the AHA definition of “ideal cardiovascular health factors” [16]. Our data, together with those of previous researches provide evidence that individuals with multiple favorable CVD risk factors clustered is uncommon in China. Our finding that the low risk profile in women is approximately 3 times of men is in line with most other studies conducted in China [21–25]. Studies from other countries have also seen a larger percentage of women with low risk profile compared with men [28–30]. In our present study, the significantly lower rates of not currently smoking and untreated systolic/diastolic BP < 120/80 mmHg among men may probably account for the gender difference in the prevalence of low risk profile.

Our finding that older individuals saw a lower prevalence of low risk profile compared with younger counterparts is also consistent with previous research [22, 23]. China has experienced rapid increase in national wealth over the past 20 years, the consequence of which is that lifestyle and the inhabitant environment (e.g. increase in energy intake) have changed dramatically. Older populations within this transitional country, who have traditionally suffered from facing famine, may eventually come to experience the highest risk for suffering from obesity, diabetes, hypertension, and so on. At the same time, China is experiencing rapidly aging population, which is expected to drastically decrease the frequency of low risk profile, too. Decreasing prevalence of low risk profile combined with the aging population indicate that urgent implementation and prevention programs in older adults should be prioritized. Furthermore, our finding that the prevalence of low risk profile decreased more rapidly among rural residents. Unfavorable trends in 3 factors contributed to this issue: BMI < 25 kg/m^2^, WC < 90/80 cm, and untreated SBP/DBP < 120/80 mmHg. Given its large population living in rural region, China may bear a relatively higher burden of CVD events. Finally, our finding that a lower prevalence of low risk profile observed in participants in the lowest education group indicates that the CVD burden also affects the lower social classes as education is a good indicator of socioeconomic status.

Obesity is the predominant factor for the development of diabetes, hypertension, and dyslipidemia [31–33]. The cause of the reductions in the prevalence of low risk profile and its components is likely related to changes in the prevalence of obesity. Despite the rapid increase in the prevalence of central and general obesity in China, previous studies have not reported the relationship of obesity status with trends in the prevalence of low risk profile and its components in China. We found that central obese persons had considerably lower prevalence of these outcomes compared with normal-WC persons. In addition, the trends in the prevalence of low risk profile and its components varied by BMI categories, with general obese individuals seeing the most dramatic change. These data suggest that increasing body weight may have fueled declines in the incidence of low risk profile and its components. Hence, urgent implementation of prevention programs in central obese and general obese persons should be prioritized and increasing attention to obesity among patients and providers may lead to increased opportunistic screening of individuals with low risk profile in health care settings.

Our study has several limitations. First, the sample is partial nationally representative as only nine of China’s 31 provinces are included, and therefore, extrapolating results to the whole of China should be interpreted cautiously. Second, other variables such as lipid profile, dietary intake, and physical activity, which have important impact on the prevalence of low risk profile, were not considered. Third, estimates across subgroups should also be interpreted with caution because of the limited sample size. Nevertheless, our study has several strengths including a vigorous quality assurance program and the same strict methodology used to ensure the quality of the data collection over the entire study period.

## Conclusions

In the present study, we found that the low risk profile and its components significantly deteriorated irrespective of gender, age groups, rural/urban settings, education groups, WC status, and BMI categories. Central obese and general obese persons desire greater concern. Given that primordial prevention holds great opportunities for decreasing the CVD burden, public health management to provide strategies for adequate risk assessment and further preventative strategies to improve the health status are urgently needed.

## Electronic supplementary material

Additional file 1: Table S1: Trends in prevalence of the 5 components of the low risk profile*. (DOC 170 KB)
