# Supplementary material for: Secular trends in the prevalence of low risk factor burden for cardiovascular disease according to obesity status among Chinese adults, 1993–2009
Source: BMC Public Health. 2014 Sep 16;14:961. doi: 10.1186/1471-2458-14-961 (PMC4189202; doi:10.1186/1471-2458-14-961)
Supplement: Supplementary file 1 — Additional file 1: Table S1: Trends in prevalence of the 5 components of the low risk profile*. (DOC 170 KB) [file 12889_2013_7096_MOESM1_ESM.doc]

# Table S1 Trends in prevalence of the 5 components of the low risk profile*

|  |  | **1993** | | **1997** | **2000** | **2004** | **2006** | **2009** | **P for trend**^†^ |
| --- | --- | --- | --- | --- | --- | --- | --- | --- | --- |
| Not currently smoking  (%) | Sex | | | | | | | | |
|  | Men | 38.4 | | 42.0 | 44.1 | 45.4 | 46.9 | 45.8 | <0.001 |
|  | Women | 95.9 | | 95.7 | 96.2 | 97.0 | 97.6 | 97.8 | <0.001 |
|  | Age (y) | | | | | | | | |
|  | 18-44 | 69.6 | | 69.8 | 71.7 | 72.8 | 74.5 | 73.8 | <0.001 |
|  | 45-64 | 64.7 | | 66.4 | 67.9 | 68.8 | 69.7 | 69.7 | <0.001 |
|  | 65-118 | 71.2 | | 76.5 | 76.0 | 76.3 | 78.4 | 75.5 | <0.001 |
|  | Rural/urban settings | | | | | | | | |
|  | Urban | 69.3 | | 70.8 | 72.8 | 72.5 | 73.8 | 74.4 | <0.001 |
|  | Rural | 68.4 | | 68.8 | 70.3 | 72.0 | 73.6 | 72.3 | <0.001 |
|  | Education | | | | | | | | |
|  | Less than high school | 68.3 | | 69.1 | 70.3 | 71.4 | 72.9 | 71.3 | <0.001 |
|  | High school | 66.3 | | 67.5 | 71.2 | 70.9 | 70.5 | 69.5 | <0.001 |
|  | University | 72.3 | | 75.5 | 75.5 | 73.2 | 77.3 | 78.9 | <0.001 |
| Body mass index <25 kg/m^2^ (%) | Sex | | | | | | | | |
|  | Men | 88.1 | | 83.5 | 79.1 | 75.9 | 73.8 | 72.4 | <0.001 |
|  | Women | 84.6 | | 81.6 | 78.0 | 76.9 | 76.9 | 77.0 | <0.001 |
|  | Age (y) | | | | | | | | |
|  | 18-44 | 89.4 | | 85.8 | 82.0 | 80.3 | 79.5 | 78.8 | <0.001 |
|  | 45-64 | 79.7 | | 75.5 | 71.3 | 68.1 | 67.0 | 66.1 | <0.001 |
|  | 65-118 | 83.7 | | 80.0 | 75.6 | 73.0 | 71.5 | 71.4 | <0.001 |
|  | Rural/urban settings | | | | | | | | |
|  | Urban | 83.4 | | 78.6 | 75.9 | 74.1 | 75.6 | 75.2 | <0.001 |
|  | Rural | 88.1 | | 85.3 | 80.6 | 78.4 | 76.2 | 75.2 | <0.001 |
|  | Education | | | | | | | | |
|  | Less than high school | 86.2 | | 83.1 | 78.8 | 76.3 | 74.6 | 74.0 | <0.001 |
|  | High school | 84.2 | | 78.7 | 77.3 | 75.7 | 76.4 | 77.0 | <0.001 |
|  | University | 88.4 | | 79.1 | 74.3 | 76.0 | 77.8 | 77.6 | <0.001 |
| Waist circumference<90 cm among men and <80 cm among women  (%) | Sex | | | | | | | | |
|  | Men | 90.8 | | 86.2 | 81.0 | 77.8 | 76.5 | 72.9 | <0.001 |
|  | Women | 72.4 | | 69.5 | 64.3 | 61.3 | 60.4 | 56.7 | <0.001 |
|  | Age (y) | | | | | | | | |
|  | 18-44 | 87.4 | | 83.7 | 79.2 | 76.1 | 75.3 | 72.1 | <0.001 |
|  | 45-64 | 71.1 | | 67.6 | 61.6 | 58.1 | 56.2 | 51.5 | <0.001 |
|  | 65-118 | 68.9 | | 64.9 | 56.5 | 54.2 | 52.7 | 49.8 | <0.001 |
|  | Rural/urban settings | | | | | | | | |
|  | Urban | 77.2 | | 74.9 | 71.5 | 68.1 | 67.6 | 65.7 | <0.001 |
|  | Rural | 84.2 | | 80.3 | 74.2 | 71.2 | 69.6 | 65.3 | <0.001 |
|  | Education | | | | | | | | |
|  | Less than high school | 80.1 | | 77.0 | 71.5 | 67.9 | 66.8 | 62.2 | <0.001 |
|  | High school | 83.3 | | 78.4 | 74.0 | 71.4 | 71.7 | 68.9 | <0.001 |
|  | University | 84.4 | | 75.9 | 74.4 | 73.3 | 71.6 | 71.6 | <0.001 |
| Untreated systolic/  diastolic blood pressure <120/80 mmHg (%) | Sex | | | | | | | | |
|  | Men | 47.7 | | 39.1 | 37.1 | 32.9 | 34.3 | 32.2 | <0.001 |
|  | Women | 60.3 | | 53.5 | 53.2 | 50.8 | 51.8 | 49.9 | <0.001 |
|  | Age (y) | | | | | | | | |
|  | 18-44 | 64.4 | 55.5 | | 55.7 | 50.3 | 52.7 | 52.8 | <0.001 |
|  | 45-64 | 42.2 | 33.4 | | 31.7 | 30.9 | 30.7 | 24.6 | <0.001 |
|  | 65-118 | 21.6 | 16.5 | | 15.6 | 17.9 | 19.1 | 12.4 | <0.001 |
|  | Rural/urban settings | | | | | | | | |
|  | Urban | 54.4 | | 47.0 | 44.7 | 43.5 | 43.8 | 43.2 | <0.001 |
|  | Rural | 56.2 | | 47.1 | 47.7 | 42.9 | 45.1 | 42.5 | <0.001 |
|  | Education | | | | | | | | |
|  | Less than high school | 52.8 | | 44.7 | 44.4 | 40.8 | 42.2 | 38.7 | <0.001 |
|  | High school | 57.7 | | 48.4 | 47.6 | 45.8 | 47.2 | 46.2 | <0.001 |
|  | University | 61.1 | | 50.1 | 48.5 | 44.8 | 47.5 | 50.3 | <0.001 |
| Not having been previously diagnosed with diabetes (%) | Sex | | | | | | | | |
|  | Men | 100.0 | | 99.6 | 99.5 | 98.9 | 98.4 | 97.9 | <0.001 |
|  | Women | 100.0 | | 99.4 | 99.1 | 98.9 | 98.6 | 98.4 | <0.001 |
|  | Age (y) | | | | | | | | |
|  | 18-44 | 100.0 | | 99.9 | 99.8 | 99.7 | 99.6 | 99.6 | <0.001 |
|  | 45-64 | 100.0 | | 99.0 | 98.9 | 98.1 | 97.4 | 96.5 | <0.001 |
|  | 65-118 | 99.9 | | 98.4 | 97.2 | 95.6 | 94.4 | 93.6 | <0.001 |
|  | Rural/urban settings | | | | | | | | |
|  | Urban | 99.9 | | 99.2 | 98.9 | 98.4 | 97.6 | 97.4 | <0.001 |
|  | Rural | 100.0 | | 99.7 | 99.6 | 99.4 | 99.2 | 98.9 | <0.001 |
|  | Education | | | | | | | | |
|  | Less than high school | 100.0 | | 99.5 | 99.3 | 99.0 | 98.4 | 98.1 | <0.001 |
|  | High school | 100.0 | | 99.5 | 99.4 | 98.9 | 98.8 | 98.9 | <0.001 |
|  | University | 100.0 | | 99.4 | 99.3 | 99.1 | 98.8 | 98.6 | <0.001 |

^*^ Estimates are weighted to be representative of the Chinese population aged 18 to 118 years.

^†^ Trends in prevalence of the 5 components of the low risk profile from 1993 to 2009 were assessed by Cochran–Armitage trend testing.
